# Aptamer-facilitated Protection of Oncolytic Virus from Neutralizing Antibodies

**DOI:** 10.1038/mtna.2014.19

**Published:** 2014-06-03

**Authors:** Darija Muharemagic, Anna Zamay, Shahrokh M Ghobadloo, Laura Evgin, Anna Savitskaya, John C Bell, Maxim V Berezovski

**Affiliations:** 1 Department of Chemistry, University of Ottawa, Ottawa, Ontario, Canada; 2 Institute of Molecular Medicine and Pathological Biochemistry, Krasnoyarsk State Medical University, Krasnoyarsk, Russia; 3 Department of Biochemistry, Microbiology and Immunology, Faculty of Medicine, University of Ottawa, Ottawa, Ontario, Canada; 4 Jennerex, San Francisco, California, USA

**Keywords:** aptamers, neutralizing antibodies, vesicular stomatitis virus

## Abstract

Oncolytic viruses promise to significantly improve current cancer treatments through their tumor-selective replication and multimodal attack against cancer cells. However, one of the biggest setbacks for oncolytic virus therapy is the intravenous delivery of the virus, as it can be cleared from the bloodstream by neutralizing antibodies before it reaches the tumor cells. We have selected DNA aptamers against an oncolytic virus, vesicular stomatitis virus, using a competitive binding approach, as well as against the antigen binding fragment (Fab) of antivesicular stomatitis virus polyclonal antibodies, in order to shield the virus from nAbs and enhance its *in vivo* survival. We used flow cytometry to identify these aptamers and evaluated their efficiency to shield vesicular stomatitis virus in a cell-based plaque forming assay. These oligonucleotides were then modified to obtain multivalent binders, which led to a decrease of viral aggregation, an increase in its infectivity and an increase in its stability in serum. The aptamers were also incubated in nondiluted serum, showing their effectiveness under conditions mimicking those *in vivo*. With this approach, we were able to increase viral infectivity by more than 70% in the presence of neutralizing antibodies. Thus, this method has the potential to enhance the delivery of vesicular stomatitis virus through the bloodstream without compromising the patient's immune system.

## Introduction

**Figure 1:**
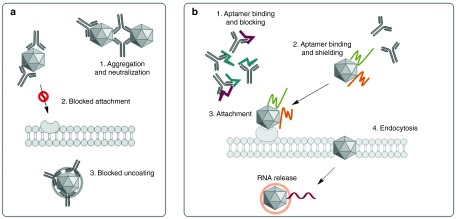
**General scheme of aptamer-facilitated protection of oncolytic virus from neutralizing antibodies**. (**a**) Anti–vesicular stomatitis virus neutralizing antibodies bind to the viruses and cause several effects: (i) aggregation, (ii) blocking attachment of the virus to the cell membrane, and (iii) preventing uncoating of the virus inside the cell. (**b**) Aptamers blocking the Fab fragments of antibodies and shielding aptamers binding to the virus prevent the neutralization of the virus, thus allowing it to infect the cell.

Oncolytic viruses (OVs) promise to significantly improve current cancer treatments through their tumor-selective replication and multimodal attack against cancer cells.^1^ A variety of OVs have been adopted or specifically engineered to selectively kill cancer cells. Some of these are currently under clinical or preclinical studies.^2^ Human trials have demonstrated that the use of OVs is safe with much less toxicity compared to standard forms of cancer therapy, such as chemo- and radiation therapy.^3^ However, one of the biggest obstacles for OV therapy is the intravenous delivery of the virus, as it can be cleared from the bloodstream before it reaches the tumor cells. The virus in the bloodstream could be inactivated by complement proteins^4,5^ or the reticuloendothelial system,^6,7^ but the most restrictive barrier to effective treatment is the acquired immunity to repeated infections due to neutralizing antibodies (nAbs) (**Figure 1a**).^8,9,10,11^ Several approaches to reduce the negative impact of adaptive immunity on OVs have been developed, such as the use OVs with cyclophosphamide as an immunosuppressive drug,^12^ polymer-coated OVs to shield the virus from its nAbs^13,14,15^ and virus-preinfected T cells or syngeneic carrier cells that act as delivery vehicles to tumor sites and shielding agents.^16,17,18,19^ Polymer coating and cell-based methodologies increase the virus stability in blood but significantly decrease the uptake of OVs by tumor cells due to blocking virus–cell receptor interactions. The last method requires patients' own T cells to be isolated, activated, and infused back to the patients. This complicated procedure makes it difficult for clinical use. Development of aptamers binding to OVs and/or nAbs seems promising as they could allow the virus to escape the host immune mechanism and neutralization.

We have previously developed anti-nAbs aptamers and showed their potential in preventing nAbs from binding to an OV and inhibiting infection with flow cytometry and plaque forming assays.^20^ Additionally, we presented the development and use of anti-vesicular stomatitis virus (VSV) antibodies and applied these oligonucleotides in antibody displacement assays using biosensors.^21^ Here, we propose an improved aptamer-based technology to enhance the survival of OVs in the presence of nAbs. The technology is called Aptamer-Facilitated Virus Protection (AptaVIP) and is based on two types of DNA aptamers: blocking and shielding aptamers. Blocking aptamers bind to antigen-binding fragments (Fab) of nAbs (anti-nAbs aptamers) and prevent neutralization of a virus; shielding aptamers bind to the virus and mask it from recognition by nAbs allowing the virus to attach to and infect cancer cells (**Figure 1b**).

In this study, we used DNA aptamers with different affinities to VSV, or to rabbit anti-VSV antibodies, selected by two modified SELEX procedures. VSV is one of the several potent OVs. It has a broad cancer cell tropism and is effective when administrated intravenously in several murine tumor models, which is hypothesized to be associated with its vulnerability to the interferon pathway.^22,23,24,25^ A mutation in the M protein of the virus (VSVΔ51) enhances this vulnerability resulting in improved tumor-cell selectivity.^26^ Plaque forming assay on cancer cells demonstrated aptamers' ability to recover viral infectivity in the presence of rabbit serum containing neutralizing anti-VSV antibodies. By modifying these aptamers in order to obtain multivalent binders, we increased infectivity up to 77% as well as elevated serum stability of the aptamers.

## Results

### Selection of anti-VSV aptamers

**Figure 2:**
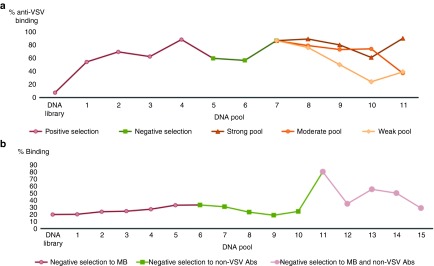
**Selection of DNA aptamers to VSV and VSV-nAbs**. (**a**) Selection of aptamers against VSV with 11 rounds of selection: (i) four positive rounds of selection, (ii) three negative rounds of selection against blood cells, and (iii) four rounds of selection using a competitive approach with a 96-well plate. Binding of resulting pools was analyzed by flow cytometry using FAM-labeled aptamer pools. (**b**) Selection of aptamers aginst VSV-nAbs with 15 rounds of selection. Each positive selection was preceded with a negative selection: (i) five negative selections against magnetic beads (MB), (ii) five rounds of negative selection against non-VSV Abs, and (iii) five rounds of selection against MB and non-VSV Abs. Binding of resulting pools was analyzed by flow cytometry using FAM-labeled aptamer pools. nAb, neutralizing antibody; VSV, vesicular stomatitis virus.

Shielding aptamers against VSV were selected by 11 rounds of the cell-SELEX procedure^21^ at 37 °C, that is schematically represented in **Supplementary Figure S1a**. Briefly, each round of selection consisted of six steps: incubation of DNA library with VSV, removal of unbound DNA from VSV, negative selection against plastic vials, separation of bound DNA sequences from virus, DNA amplification by symmetric and asymmetric polymerase chain reaction (PCR), and purification of single-stranded aptamers after PCR. Consequently, after first four positive rounds of selection, three rounds of negative selection against human blood cells, mouse blood cells, and plasma were performed. These negative rounds of selection resulted in a decrease of overall affinity, as is shown in **Figure 2a** (pools 5 and 6), due to the removal of sequences that bound to both the virus and nonspecific targets.

Next selection step was performed using a competitive approach, schematically represented in **Supplementary Figure S1b**. A nonlabeled aptamer pool with an affinity for VSV was incubated in a 96-well plate in order to allow DNA to nonspecifically adhere to the plastic. Then, virus particles, previously incubated with an anti-VSV aptamer pool, were added to the well. Adhesion of the virus onto the plate was verified with an intercalating viral RNA dye, YOYO-1, and analyzed by a fluorescence plate reader. Finally, the sandwich-like complex of aptamers and virus was disrupted by addition of antibodies at three concentrations: low, moderate, and high (0.5, 2, and 3 µg/ml). Aptamers that were displaced from the surface of the virus with these different antibody concentrations were separately collected, amplified, and used for further selection.

To summarize, we performed 11 selection rounds and monitored the enrichment using fluorescently labeled pools of aptamers, incubated them with VSV, and analyzed their binding by flow cytometry. Gating strategy for the OV in flow experiments is shown in **Supplementary Figure S2**. An increase of binding affinity of the strong pool to VSV was observed during the competitive selection on plate. Binding of the moderate pool did not significantly increase until the tenth round. Furthermore, we observed a decrease of affinity for the weak pool during the competitive selection. However, in round 11, this pool had a higher affinity for the target than the moderate pool (**Figure 2a**).

### Displacement analysis by neutralizing antibodies

**Figure 3:**
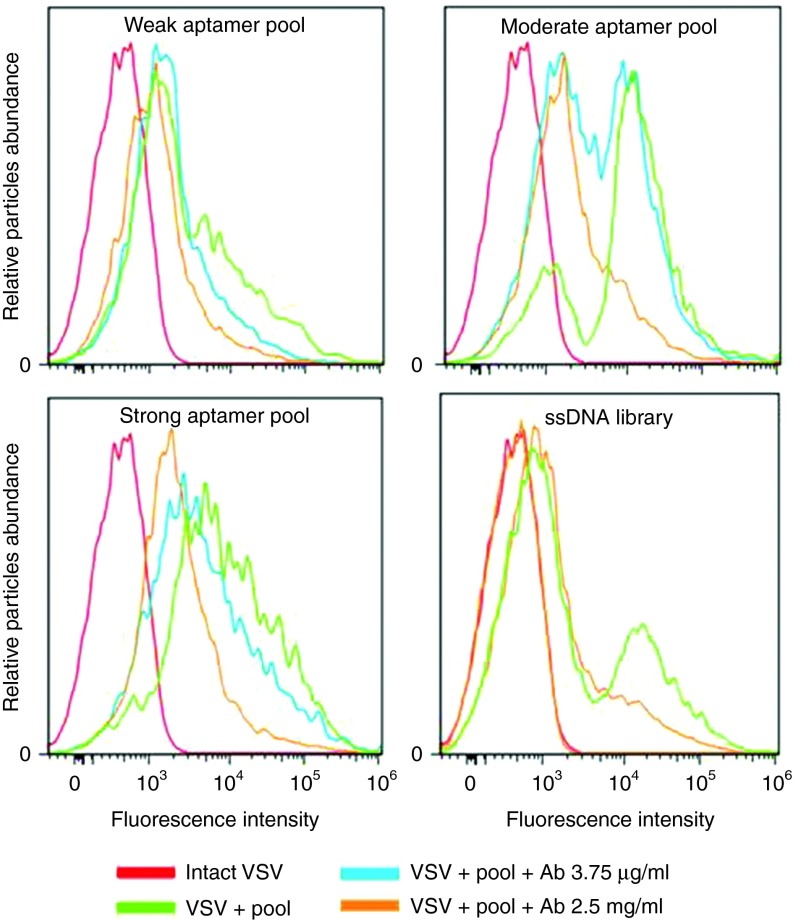
**Flow cytometry binding and competitive analysis of aptamer pools**. Flow cytometric analysis of competitive binding between FAM-labeled weak, moderate and strong aptamer pools (200 nmol/l) and VSV (1 × 10^7^ PFU), followed by introduction of anti-VSV nAbs. Red curve represents virus alone, which was considered as a negative control. Green curve corresponds to the VSV binding with aptamer pools, or ssDNA library without anti-VSV nAbs. Blue and orange curves show the binding after incubation with anti-VSV nAbs (3.75 µg/ml or 2.5 mg/ml, respectively). nAb, neutralizing antibody; VSV, vesicular stomatitis virus.

All weak, moderate, and strong aptamer pools were analyzed for their ability to bind the virus and be displaced by anti-VSV nAbs. FAM-labeled (6-Carboxy-fluorescein) aptamers were preincubated with virus and analyzed by flow cytometry. Neutralizing anti-VSV antibodies were then added to the mixture and incubated an additional hour at 37 °C. The displacement of aptamers from the surface was estimated by monitoring the reduction of virus fluorescence. The best pools corresponding to weak, moderate, and strong aptamers are presented in **Figure 3**. Weak pool 11 was displaced from viral surface with antibodies (3.8 µg/ml). Further addition of antibodies at a high concentration (2.5 mg/ml) did not significantly change the fluorescence, suggesting that aptamers that shared the same binding sites with antibodies had been replaced with the lower concentrations of nAbs. Moderate pool 10 was partially displaced with the antibody at a concentration of 3.8 µg/ml, and the additional dose of antibody of 2.5 mg/ml led to further reduction of fluorescence. Strong pool 11 had a high binding affinity and was not displaced significantly with antibodies at a concentration of 3.8 µg/ml. However, the high concentration of antibodies shifted the fluorescence curve back to the same intensity as the virus alone, indicating that nearly all aptamers bound to VSV had been replaced. Finally, moderate pool 10 and strong pool 11 were cloned (**Table 1**). The obtained clones were sequenced and then grouped based on conserved sequence motifs that were discovered using Clustal Omega software (**Supplementary Table S1**). The EC_50_ (half maximal effective concentration) for the strong pool 11 determined by flow cytometry was 80 nmol/l.

### Selection of anti-nAbs aptamers

Selection of blocking aptamers to Fab fragments of nAbs was previously reported by our group.^20^ Briefly, aptamer pools were selected using protein G-coated magnetic beads that were coupled with polyclonal antibodies from a rabbit previously immunized against VSV. A series of negative selections were done against the magnetic beads alone and magnetic beads coupled with antibodies not specific to VSV obtained from nonimmunized rabbit's serum. Aptamers were analyzed by flow cytometry and the pool showing the highest affinity and the most effective shielding was cloned and sequenced. These clones were further analyzed in a competitive binding assay, where DyLight 488-conjugated anti-VSV nAbs were preincubated with the aptamers and then added to the VSV. Fluorescence of the virus–antibody complex was assessed by flow cytometry. A decrease of fluorescence indicated that there was a competition between the aptamers and virus for binding the antibodies.

### Analysis of shielding and blocking aptamers by plaque forming assays

The ability of individual aptamer clones to protect the virus from nAbs was tested by a plaque forming assay in Vero cells. In order to detect plaques in this assay, yellow fluorescent protein (YFP)-expressing VSV was used. To quantify the protection effect, plaques were counted and virus infectivity calculated. The virus sample without nAbs was considered to have 100% infectivity, and the virus with nAbs and without any plaque formation shows 0% infectivity.

Shielding properties of individual anti-VSV aptamer sequences from moderate and strong pools, as well as aptamer sequences that were previously selected in our lab,^26^ were tested. We found that the clone Z-23 had the ability to significantly prevent the neutralization of virus by anti-VSV antibodies, and clones Z-22, S-31 (selected from the strong aptamer pool) and M-50 (selected from the moderate aptamer pool) also showed a shielding effect (**Supplementary Figure S4**). As for anti-nAbs aptamers, three clones (C5s, C7s, and C9) proved to have the highest blocking efficacy. To determine the optimum concentration of aptamers for the plaque forming assay, we performed a titration experiment. Both VSV and anti-VSV antibodies were incubated with their respective aptamer pools varying from 1 to 10 µmol/l concentrations. Two of the highest concentrations (1 and 10 µmol/l) showed the highest potency, whereas the shielding effect of aptamer concentrations below 10 nmol/l significantly dropped (**Supplementary Figure S5**).

**Figure 4:**
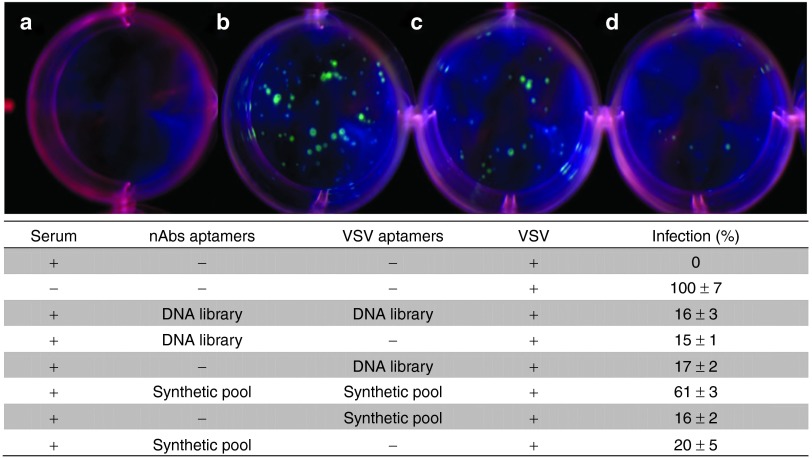
**Cell-based viral infectivity assay**. Neutralizing antibodies (nAbs) were preincubated with or without 1 µmol/l of anti-nAbs aptamers. The complex was then incubated with vesicular stomatitis virus (VSV) preincubated with anti-VSV aptamers and 100 PFUs were added to Vero cells in a 12-well plate. (**a**) Cells infected with VSV in the presence of nAbs (0% infectivity); (**b**) Cells infected with VSV without nAbs (100% infectivity); (**c**) Cells infected with VSV in the presence of nAbs, and anti-nAbs and anti-VSV aptamer pools; and (**d**) Cells infected with VSV in the presence of nAbs and DNA Library as a control experiment. Results of additional control experiments are presented in the adjusted table. All plaque forming assays were performed in triplicates.

It is interesting to note that pools for anti-VSV and anti-nAbs aptamers tested individually showed an increase of infection by 20% (**Figure 4**). The combination of pools for both targets resulted in the highest increase, with 61% additional plaques, suggesting a synergistic mechanism. On average, 30% of plaques were formed from infection of cells with virus when it was incubated with the native DNA library as a nonspecific control.

### Effect of dimeric and tetrameric aptamers on VSV infectivity

**Figure 5:**
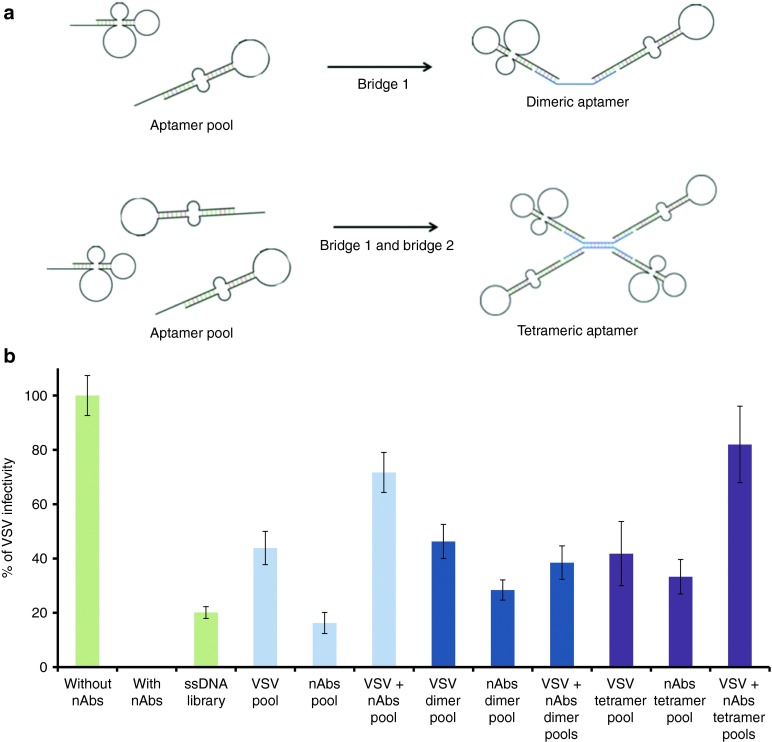
**Dimeric and tetrameric aptamers**. (**a**) General scheme of construction of dimers and tetramers using bridge 1, or bridges 1 and 2, respectively. (**b**) Plaque forming assay results showing the infectivity of VSV using unmodified aptamer pools, dimer pools or tetramer pools for VSV, nAbs, or both VSV and nAbs. Plaque forming assays were performed in triplicates. nAb, neutralizing antibody; VSV, vesicular stomatitis virus.

In order to increase the potency of our aptamers, we tested the possibility of using multivalent aptamers. For this purpose, we constructed dimeric and tetrameric aptamers, linked by an oligonucleotide bridge (**Figure 5a**). The apparent dissociation constant for monomeric and dimeric pools remained similar (71 ± 15 and 87 ± 6 nmol/l, respectively), whereas a fourfold decrease for the tetrameric pool (22 ± 9 nmol/l) was observed. With the use of anti-VSV dimers and tetramers, virus infectivity with VSV aptamers remained approximately the same, with a difference of ±2%. However, the use of dimeric and tetreameric forms for anti-nAbs aptamers resulted in an increase by 12 and 28% of viral infectivity, respectively. The combination of aptamer pools in a dimeric form did not have an increasing effect on VSV infectivity. Conversely, this pool combination with a tetrameric bridge increased the infectivity to 77% (**Figure 5b**).

**Figure 6:**
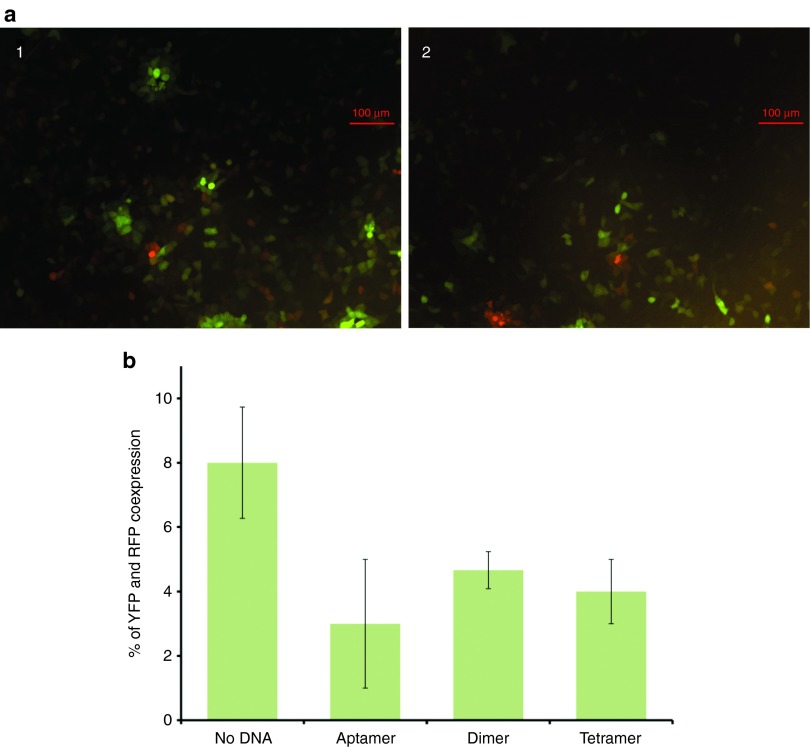
**Fluorescence microscopy aggregation assay**. Aptamers in monomeric, dimeric, and tetrameric form were incubated in equal amounts of VSV-YFP and VSV-RFP and used to infect a monolayer of Vero cells. (**a**) Overlay of YFP and RFP expression of cells infected with virus incubated (i) without the aptamers and (ii) with tetrameric aptamers. Cells expressing YFP (green), RFP (red), or both (orange) were counted. (**b**) Cells infected with virus alone, virus with monomeric, dimeric, and tetrameric aptamers showed coexpression of both YFP and RFP in 8, 3, 5, and 4% of all cells, respectively. VSV, vesicular stomatitis virus.

Finally, we did an aggregation assay where a mixture of VSV-YFP and VSV-RFP (red fluorescent protein) was incubated either without aptamers, or with monomeric, dimeric or tetrameric aptamers. Following the plaque forming assay, the plaques were visualized by fluorescence microscopy in order to detect the cells expressing YFP and RFP. We assumed that the percentage of cells expressing both proteins correlates with viral aggregation, as it indicates that a cell has been infected by more than one virus. This assay showed that both YFP and RFP were expressed in 8, 3, 5, and 4% of cells infected with virus alone and virus with dimeric, monomeric, and tetrameric aptamers, respectively (**Figure 6**).

## Discussion

Viral therapy is a promising oncolytic tool as it allows selective targeting of cancer cells with an onset of relatively mild adverse reactions to the system. However, tumor infection *in vivo* remains inefficient due to the body's antiviral response, which leads to a production of nAbs.^4^ Aptamers have previously been reported as successful inhibitors against enzymes, as well as antiviral agents with high binding affinities to their respective targets.^27,28^ Therefore, we selected aptamers against VSV and anti-VSV, hypothesizing that binding of these will reduce the interaction between the two targets and thus promote a shielding effect for the virus (**Figure 1**).

The initial aptamer selection was performed on the basis of the Systematic Evolution of Ligands by Exponential Enrichment (SELEX) technology^29,30^ from native ssDNA library, which had a randomized region of 40 nucleotides, ﬂanked by two primer binding sites. Pools resulting from negative selections demonstrated a decreased overall affinity, which led us to believe that the number of sequences had significantly decreased as well, potentially increasing the specificity of the DNA pool to infectious VSV.

Consequent selection steps consisted of a competitive approach. We hypothesized that the addition of different concentrations of antibodies would allow us to collect aptamers that (i) share the same binding site as the antibodies and (ii) have different dissociation constants for their targets. Effectively, the hypothesis correlated with the analysis of these pools by flow cytometry, where we observed the displacement of aptamers from the weak pool with the addition of low antibody concentrations. Conversely, in order to displace aptamers from the strong pool, a high concentration of antibodies was necessary, suggesting that these aptamers bind with a much higher affinity to the target.

As with most cancer cell lines, VSV infects Vero cells, lyses them, and proliferates to neighboring cells.^31^ In the presence of nAbs, this virus is inactivated and thus unable to infect the monolayer of cells. We first tested all the aptamer clones and ensured that they did not reduce the infectivity of the virus. It is likely that given aptamers' much smaller size, compared to nAbs, they do not interfere with VSV binding and uptake. However, when we incubated, either the antibodies or the virus with their cognate aptamers, we achieved an increase of viral infectivity in the presence of nAbs. Interestingly, VSV-binding clones that had an efficient shielding effect did not only come from the strong pool that was obtained from competitive selection approach, but also from the medium pool as well as from a pool that was selected in our previous work, using a standard cell-SELEX method.^25^

Aptamers binding to nAbs were incubated with whole, nondiluted rabbit serum for 5 minutes. The short incubation time imitated more closely the effect of aptamers being introduced to blood-circulating antibodies. Pools for nAbs or VSV tested individually showed an increase of infection of 20%. The combination of pools for both targets resulted in the highest increase, with 61% of additional plaques. On average, 32% of plaques were formed from infection of cells with virus incubated with the native DNA library (**Figure 4**). This effect lead us to believe that due to the polyclonal nature of the antibodies, it is better to have a large number of aptamers that could bind nAb variants. Therefore, the best results are obtained when combining pools to achieve higher specificity and a larger number of different aptamers.

Due to the instability of aptamers in serum, we modified our oligonucleotides by constructing their dimeric and tetrameric counterparts. These were constructed by bridging monomers using a single- or double-stranded DNA bridge, respectively (**Figure 5a**). Each strand consisted of extremities that were complementary to forward and reverse primer-binding domains of aptamers. Some primer regions can participate in the folding of our aptamers, therefore having two different binding sites decreases the risk of disrupting the secondary structure that may be important in binding the target. For these plaque forming assays, nondiluted serum was used in order to mimic the effect of aptamers interacting with other components present in serum and undergoing degradation by nucleases.^32^ We have also shown that these multivalent structures decrease the aggregation of the VSV, which may contribute to increasing its infectivity (manuscript in preparation). Furthermore, our degradation experiments showed that the dimeric and tetrameric forms of the aptamers are more stable in serum (**Supplementary Figure S6**). We analyzed the amount of aptamer in serum using quantitative PCR, and based on the standard curve, there was about five times less of the monomeric form after a 24-hour incubation period in serum (**Supplementary Figure S6a**). However, investigating the melting curve of various samples indicates that this loss is at least two times greater. It is clear that a significant portion of amplified products consists of smaller, degraded aptamers (**Supplementary Figure S6b**). We hypothesize that this is due to the fact that the aptamers became less accessible to exonucleases. Finally, the aggregation assay showed a decrease of aggregation when incubated with dimers and tetramers, suggesting a repulsion of viral particles caused by the negatively charged nucleotides, which could also interfere with viral neutralization caused by anti-VSV antibodies.

In this proof-of-concept article, we have demonstrated the use of aptamers in order to block anti-VSV antibodies and shield the VSV from antibody neutralization. Furthermore, we have modified these aptamers by bridging them together to form tetrameric counterparts, and were able to achieve 77% viral infectivity in a plaque forming assay. These types of aptamers can potentially be applied *in vivo*, in combination with the OV, to prevent clearance of the virus and thus increase its oncolytic efficiency.

## Materials and methods

*Materials.* All oligonucleotides were purchased from Integrated DNA Technologies (IDT, Coralville, IA). All media and buffers were purchased from Sigma-Aldrich (St Louis, MO), unless otherwise stated.

Original VSV sample was provided by Jennerex (San Francisco, CA). It was then propagated and purified as described by Diallo *et al*.^33^ Briefly, Vero African green monkey kidney cells were seeded in High glucose Dulbecco's modified Eagle medium supplemented with 10% fetal bovine serum. When cultures reached about 95% confluency, they were infected with the virus at a concentration of 2 × 10^5^ plaque forming units (PFUs) per 150-mm petri dish. After ~24 hours, the supernatant containing the virus was collected and purified by centrifugation and sucrose gradient. Aliquoted viral samples were titered and stored at −80 °C. Rabbit serum containing polyclonal anti-VSV antibodies (~50 mg/ml) was provided by Jennerex and stored at −20 °C.

*Selection of anti-VSV aptamers.* Briefly, four rounds of selection against intact VSV were performed at 37 °C using the protocol schematically presented in **Supplementary Figure S1a** and provided in details in our previous publication.^21^ Briefly, each round of the selection consists of six consecutive steps: (i) incubation of single-stranded (ss) DNA library, having a randomized region of 40 nucleotides ﬂanked by two primer-hybridization sites, with VSV, (ii) partitioning of unbound DNA from VSV, (iii) negative selection to plastic vials, (iv) extraction of bound DNA sequences, (v) DNA amplification by symmetric and asymmetric PCR, and (vi) purification of ssDNA aptamers from PCR products. To monitor the enrichment, fluorescently labeled pools of aptamers were incubated with VSV and analyzed by flow cytometry. The binding of the pools to VSV was found to increase from rounds 1 to 4, where saturation was reached. Finally, 100 nmol/l of the evolved aptamer pool was utilized for the next round of selection following the above-mentioned procedure.

For *in vivo* applications of aptamers against VSV, it is necessary to prepare aptamer pools that do not bind to blood cells and plasma proteins and are stable and active in blood stream. Also there is no need to protect inactivated virus particles that are not able to infect cancer cells from the immune system. Therefore, after 10 positive rounds of selection the subsequent negative selection against human and mouse blood cells and plasma and heat-treated VSV was introduced (**Supplementary Figure S2a**). The best aptamer pool, as determined by the binding assay, was incubated with heat-treated virus and unbound aptamers were collected. These were incubated with human blood cells at 37 °C for 30 minutes and unbound sequences were again collected. In the next step, the resulting pool was incubated with human blood plasma for 5 minutes and then VSV particles were added directly to this mixture and incubated for another 15 minutes. By doing this, we allowed virus to compete with plasma proteins, as well as selecting those sequences that could be stable and active in plasma. Aptamers bound to the virus were collected and the same negative selection procedure was done with the mouse blood cells and plasma. Finally aptamer sequences left after negative selection were amplified. In total, we performed 11 rounds of selection.

*Displacement selection.* A nonpurified and unlabeled aptamer pool (concentration about 200 nmol/l) with high affinity to VSV was incubated overnight in black polypropylene 96-well plates (Corning, Corning, NY). The following day, wells were washed once with Dulbecco's phosphate-buffered saline (DPBS) and then incubated with 1 × 10^7^ PFUs of VSV for 0.5 hours at 37 °C. Unbound virus particles were washed from the well with DPBS and virus that was left attached to the plate was incubated with FAM-labeled aptamer pool (**Supplementary Figure S1b**) for 1 hour at 37 °C. For the competitive plate selection, three different fractions of rabbit serum containing polyclonal anti-VSV antibodies were added to three different wells, low (0.5 µg/ml), medium (2 µg/ml), and high (3 µg/ml) and incubated for 5, 30, and 60 minutes, respectively. Following the addition of each antibody fraction, the supernatant was collected, which contained released DNA. Collected fractions of released aptamers were amplified by PCR and used for a subsequent round of selection on a plate.

*Selection of anti-nAbs aptamers.* Selection against anti-VSV rabbit antibodies was previously reported. Briefly, the selection was done using protein G-coated magnetic beads. For the first five rounds of selection, 5 × 10^5^ beads coupled with anti-VSV nAbs were incubated with 200 nmol/l of ssDNA for 1 hour at room temperature and continuous mixing. Bound DNA was separated from unbound and eluted from the beads by denaturing at 85 °C for 10 minutes. For the first five rounds, a positive selection was followed by a negative selection step where the eluted DNA was mixed with the beads alone and bound DNA was discarded. In rounds 6 to 10, the beads for the negative selection step were coupled with non-VSV antibodies prior to their incubation with the eluted DNA. In rounds 11 to 15, two negative selections, beads alone and beads with nonspecific antibodies, respectively, preceded the positive selection step. Then, unbound DNA was collected and used for incubation with anti-VSV nAbs coupled with magnetic beads. Affinity of aptamers to nAb-coated beads during the selection was monitored using flow cytometry. Selected aptamer pools were then subjected to competitive binding analysis, where 200 nmol/l of aptamers were incubated with 10 ng/µl DyLight 488-conjugated anti-VSV antibodies (Rockland, Immunochemicals, Boyertown, PA) for 1 hour, followed by addition of 6 × 10^8^ PFUs of VSV. Fluorescence of the virus was monitored by flow cytometry. Pool 11 was selected as the one having the highest affinity and the best shielding effect. Consequently, it was cloned and sequenced.

*Flow cytometric analysis.* The affinity of evolved VSV aptamer pools toward VSV was determined using FC-500 flow cytometer (Beckman Coulter, Pasadena, CA). VSV particles, 1 × 10^7^ PFUs, were preincubated with 0.1 mg/ml baker's yeast RNA (Rockland, Immunochemicals) as a masking single-stranded nucleic acid to supress nonspecific binding for 20 minutes at room temperature. Afterward, VSV particles were incubated with 100 nmol/l purified FAM-labeled aptamer pool in 50 µl DPBS buffer for 30 minutes at 25 °C. Control experiments were performed using fluorescently labeled ssDNA library in DPBS. Subsequently, each sample was washed once with 200 ml of DPBS to remove unbound DNA, resuspended in 0.5 ml of DPBS and measured. Selected aptamer pools were cloned using Perfectly Blunt Cloning Kit (Novagen, EMD Millipore, Germany) and sequenced at McGill University and Génome Québec Innovation Centre, as it has been described beforehand in our publications.^21,34^

*Displacement analysis by neutralizing antibodies.* VSV (1 × 10^7^ PFUs) was preincubated with 0.1 mg/ml yeast RNA in 50 µl DPBS for 30 minutes, at 25 °C. Afterward, it was incubated with 50 µl of 200 nmol/l FAM-labeled amplified clones, previously purified from the PCR mixture, in DPBS for 30 minutes at 25 °C. Subsequently, samples were centrifuged to remove the unbound aptamers, resuspended in 300 µl DPBS and subjected to flow cytometry analysis. After the analysis, anti-VSV antibody was added to the same vials in a final concentration of 2 mg/ml and the mixture was incubated again for 60 minutes at 37 °C. Finally, the mixture was centrifuged to remove the unbound antibodies and the replaced aptamers. VSV was resuspended in 200 µl DPBS and subjected to the second flow cytometry analysis.

*96-well plate assays.* For the neutralizing antibody assay, a procedure published by Diallo *et al*.^33^ was followed. Briefly, Vero cells were plated at a density of 1.25 × 10^4^ cells/well in 100 µl of Dulbecco's modified Eagle medium containing 10% fetal bovine serum and incubated overnight at 37 °C. The following day, rabbit serum containing anti-VSV antibodies was prepared in seven different dilutions: 100×, 500×, 1000×, 1500×, 2000×, 2500×, and 5000×. Subsequently, 67.7 µl of each concentration (performed in triplicates) was plated to a 96-well plate, to which 1 × 10^4^ PFUs of virus was added and incubated at 37 °C for 1 hour. The mixture was then transferred onto Vero cells and incubated overnight at 37 °C. The infection of cells was monitored by fluorescence using FluorChem Q (Alpha Innotech, San Leandro, CA) imaging system.

For VSV aptamer assays on 96-well plates, 1 × 10^4^ PFUs YFP-VSV were coated with aptamers at five different concentrations (100, 250, 500, 1, and 10 µmol/l) at 37 °C for 1 hour. Coated virus was then added to a 2,000× dilution of rabbit serum with anti-VSV antibodies and incubated at 37 °C for 1 hour. The remaining procedure was the same as mentioned above, where the mixture was added onto Vero cells, incubated overnight, and finally imaged in order to observe the fluorescence.

*Screening of aptamer clones and analysis of shielding and blocking aptamers by plaque forming assays.* For the plaque forming assay, Vero cells were plated at a density of 2.5 × 10^5^ cells/well in 1 ml of Dulbecco's modified Eagle medium containing 10% fetal bovine serum and incubated overnight at 37 °C. The following day, 1 × 10^4^ PFU YFP-VSV were incubated with or without VSV aptamers (final concentration 1 µmol/l) in serum-free medium for 1 hour at 37 °C. For the screening of aptamer clones, a 500× dilution of serum was used (**Supplementary Figure S3**) and incubated with or without anti-nAbs aptamers in serum-free medium for 1 hour. Serum and virus were then mixed together and placed at 37 °C for 1 hour. The mixture was diluted in order to obtain three different concentrations of virus: 100, 500, and 1,000 PFUs in 250 µl, and added to Vero cells. After 1 hour of incubation, a layer of 0.5% low-melting point agarose (IBI Scientific, Peosta, IA) with Dulbecco's modified Eagle medium supplemented with 10% fetal bovine serum was added and the plates were left to incubate for 24 hours. The following day, the plates were imaged and the plaques were counted.

Once we determined which clones were efficient candidates, the remaining plaque forming assays were performed with nondiluted rabbit serum and for a shorter time (**Figures 4** and **5**), where whole rabbit serum containing anti-VSV antibodies was incubated with or without anti-VSV aptamers (final concentration 1 µmol/l) for 5 minutes at 37 °C.

*Effect of dimeric and tetrameric aptamers on VSV infectivity.* For dimeric and tetrameric forms of aptamers, an oligonucleotide bridge linking two or four aptamers together was constructed, which consisted of one or two oligonucleotide strands connected by a central complementary sequence. Each end of the bridge consisted of a complementary flank allowing the annealing of an aptamer. To prepare the dimeric and tetrameric aptamers, after heating at 95 °C, the sides of the bridge were mixed equally, followed by their mixing with the aptamer pool in equimolar amounts. Subsequently, the complex resulted in four aptamers annealing to each bridge construct. For plaque forming assays, oligonucleotides were incubated with nondiluted serum or 1 × 10^4^ PFU of VSV, or both for 5 minutes. The serum and the virus were then mixed together and incubated for one hour at 37 °C. The virus was then diluted to 100 PFUs and added to the monolayer of Vero cells in a 12-well plate.

To test the stability of these different aptamer constructs, 20 µl of 100 µmol/l of each aptamer construct was incubated in 1 ml of human serum (BioReclamation, Hicksville, NY) at 37 °C and aliquoted during a 24-hour period. Samples were stored in −80 °C until they could be analyzed by quantitative PCR (Bio-Rad CFX; Bio-Rad Laboratories, Hercules, CA) using Platinum SYBR Green qPCR supermix-UDG reagent (Invitrogen, Carlsbad, CA) and aptamer-specific primers. Each sample was diluted in ddH_2_O and 10^4^ molecules were added to the qPCR supermix. The amplification reaction was monitored with the following thermocycler conditions: 50 °C for 2 minutes, 95 °C for 2 minutes, 40 cycles at 95 °C for 15 seconds, and 60 °C for 30 seconds. The amount of aptamers in serum was measured on the basis of a standard curve obtained from a serial dilution of aptamers in serum at 0 hour incubation (**Supplementary Figure S6**).

For the aggregation assay, Vero cells were plated in a 96-well plate at a density of 1.25 × 10^4^ cells/well. Aptamers in monomeric, dimeric or tetrameric form (4 µmol/l) were incubated with premixed equimolar solution of VSV-YFP and VSV-RFP for 1 hour at 37 °C. Following this incubation, the mixture was diluted and 25 PFUs were added to the cells and incubated at 37 °C. After 10 hours, the cells were visualized by confocal microscopy (Eclipse Ti, Nikon Corporation, Tokyo, Japan) and the number of infected cells expressing YFP, RFP, or both, were counted (**Figure 6**).

**SUPPLEMENTARY MATERIAL** **Figure S1.** Selection of anti-VSV aptamers. **Figure S2.** Gating strategy for the oncolytic virus for flow cytometry analysis. **Figure S3.** Determination of anti-VSV Ab concentration with 96-well plate assay. **Figure S4.** Screening of anti-VSV aptamers in a 96-well plate. **Figure S5.** Titration experiments showing the effect of different aptamer concentrations on VSV infectivity. **Figure S6.** Real-time PCR (qPCR) showing degradation of monomeric, dimeric, and tetrameric aptamers. **Table S1.** DNA sequences from competitive binding selection grouped into families of related sequences.

## Figures and Tables

**Table 1:**
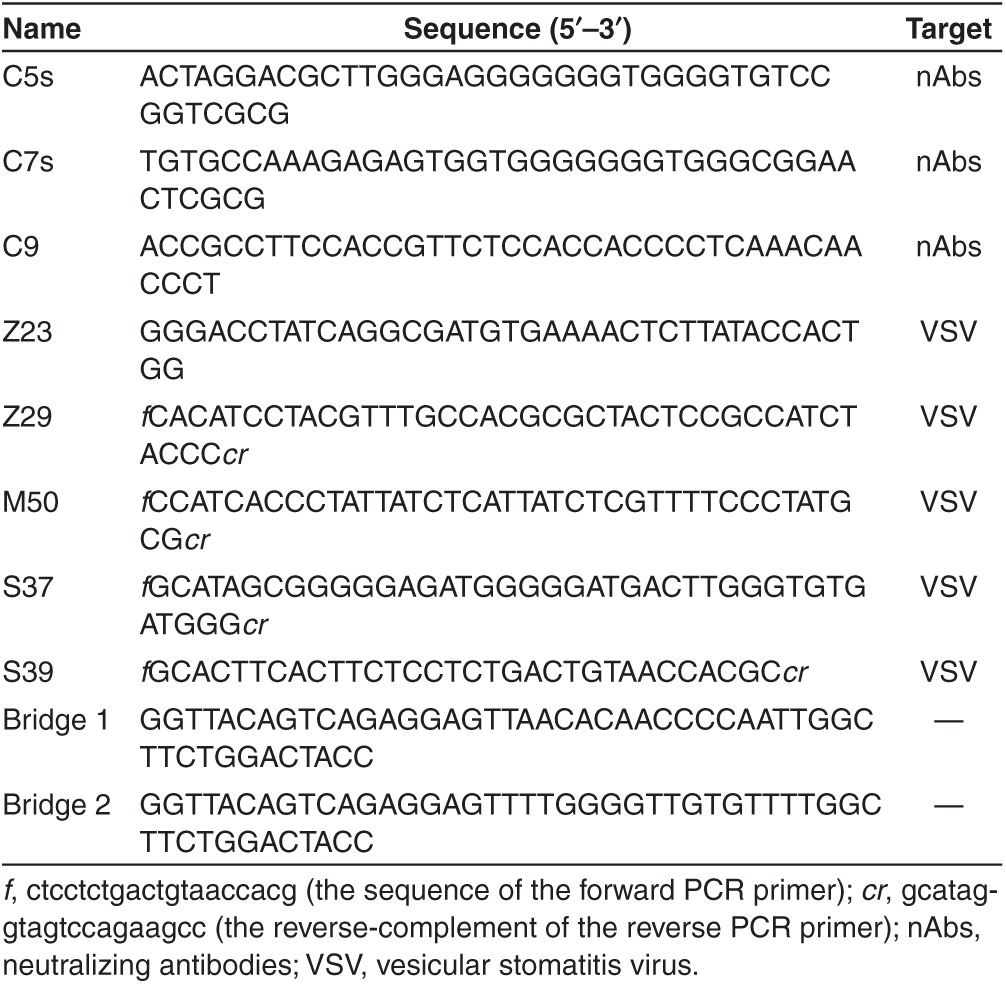
Aptamer sequences for anti-VSV neutralizing antibodies and VSV, and bridge sequences

## Supplementary Material

Supplementary Figure S1 Selection of anti-VSV aptamers.

Supplementary Figure S2 Gating strategy for the oncolytic virus for flow cytometry analysis.

Supplementary Figure S3 Determination of anti-VSV Ab concentration with 96-well plate assay.

Supplementary Figure S4 Screening of anti-VSV aptamers in a 96-well plate.

Supplementary Figure S5 Titration experiments showing the effect of different aptamer concentrations on VSV infectivity.

Supplementary Figure S6 Real-time PCR (qPCR) showing degradation of monomeric, dimeric, and tetrameric aptamers.

Supplementary Table S1 DNA sequences from competitive binding selection grouped into families of related sequences.

## References

[bib1] Breitbach CJ Burke J Jonker D Stephenson J Haas AR Chow LQ 2011 Intravenous delivery of a multi-mechanistic cancer-targeted oncolytic poxvirus in humans Nature 477 99 102 21886163 10.1038/nature10358

[bib2] Parato KA Senger D Forsyth PA Bell JC 2005 Recent progress in the battle between oncolytic viruses and tumours Nat Rev Cancer 5 965 976 16294217 10.1038/nrc1750

[bib3] Liu TC Hwang T Park BH Bell J Kirn DH 2008 The targeted oncolytic poxvirus JX-594 demonstrates antitumoral, antivascular, and anti-HBV activities in patients with hepatocellular carcinoma Mol Ther 16 1637 1642 18628758 10.1038/mt.2008.143

[bib4] Ikeda K Ichikawa T Wakimoto H Silver JS Deisboeck TS Finkelstein D 1999 Oncolytic virus therapy of multiple tumors in the brain requires suppression of innate and elicited antiviral responses Nat Med 5 881 887 10426310 10.1038/11320

[bib5] Wakimoto H Ikeda K Abe T Ichikawa T Hochberg FH Ezekowitz RA 2002 The complement response against an oncolytic virus is species-specific in its activation pathways Mol Ther 5 275 282 11863417 10.1006/mthe.2002.0547

[bib6] Worgall S Wolff G Falck-Pedersen E Crystal RG 1997 Innate immune mechanisms dominate elimination of adenoviral vectors following *in vivo* administration Hum Gene Ther 8 37 44 8989993 10.1089/hum.1997.8.1-37

[bib7] Ye X Jerebtsova M Ray PE 2000 Liver bypass significantly increases the transduction efficiency of recombinant adenoviral vectors in the lung, intestine, and kidney Hum Gene Ther 11 621 627 10724040 10.1089/10430340050015806

[bib8] Hirasawa K Nishikawa SG Norman KL Coffey MC Thompson BG Yoon CS 2003 Systemic reovirus therapy of metastatic cancer in immune-competent mice Cancer Res 63 348 353 12543787

[bib9] Lang SI Giese NA Rommelaere J Dinsart C Cornelis JJ 2006 Humoral immune responses against minute virus of mice vectors J Gene Med 8 1141 1150 16800041 10.1002/jgm.940

[bib10] Chen Y Yu DC Charlton D Henderson DR 2000 Pre-existent adenovirus antibody inhibits systemic toxicity and antitumor activity of CN706 in the nude mouse LNCaP xenograft model: implications and proposals for human therapy Hum Gene Ther 11 1553 1567 10945769 10.1089/10430340050083289

[bib11] Tsai V Johnson DE Rahman A Wen SF LaFace D Philopena J 2004 Impact of human neutralizing antibodies on antitumor efficacy of an oncolytic adenovirus in a murine model Clin Cancer Res 10 7199 7206 15534093 10.1158/1078-0432.CCR-04-0765

[bib12] Kottke T Pulido J Thompson J Sanchez-Perez L Chong H Calderwood SK 2009 Antitumor immunity can be uncoupled from autoimmunity following heat shock protein 70-mediated inflammatory killing of normal pancreas Cancer Res 69 7767 7774 19738045 10.1158/0008-5472.CAN-09-1597 PMC3046769

[bib13] Morrison J Briggs SS Green N Fisher K Subr V Ulbrich K 2008 Virotherapy of ovarian cancer with polymer-cloaked adenovirus retargeted to the epidermal growth factor receptor Mol Ther 16 244 251 18071336 10.1038/sj.mt.6300363

[bib14] Fisher KD Stallwood Y Green NK Ulbrich K Mautner V Seymour LW 2001 Polymer-coated adenovirus permits efficient retargeting and evades neutralising antibodies Gene Ther 8 341 348 11313809 10.1038/sj.gt.3301389

[bib15] O'Riordan CR Lachapelle A Delgado C Parkes V Wadsworth SC Smith AE 1999 PEGylation of adenovirus with retention of infectivity and protection from neutralizing antibody *in vitro* and in vivo Hum Gene Ther 10 1349 1358 10365665 10.1089/10430349950018021

[bib16] García-Castro J Martínez-Palacio J Lillo R García-Sánchez F Alemany R Madero L 2005 Tumor cells as cellular vehicles to deliver gene therapies to metastatic tumors Cancer Gene Ther 12 341 349 15650763 10.1038/sj.cgt.7700801

[bib17] Coukos G Makrigiannakis A Kang EH Caparelli D Benjamin I Kaiser LR 1999 Use of carrier cells to deliver a replication-selective herpes simplex virus-1 mutant for the intraperitoneal therapy of epithelial ovarian cancer Clin Cancer Res 5 1523 1537 10389942

[bib18] Power AT Wang J Falls TJ Paterson JM Parato KA Lichty BD 2007 Carrier cell-based delivery of an oncolytic virus circumvents antiviral immunity Mol Ther 15 123 130 17164783 10.1038/sj.mt.6300039

[bib19] Ong HT Hasegawa K Dietz AB Russell SJ Peng KW 2007 Evaluation of T cells as carriers for systemic measles virotherapy in the presence of antiviral antibodies Gene Ther 14 324 333 17051248 10.1038/sj.gt.3302880

[bib20] Muharemagic D Labib M Ghobadloo SM Zamay AS Bell JC Berezovski MV 2012 Anti-Fab aptamers for shielding virus from neutralizing antibodies J Am Chem Soc 134 17168 17177 23016897 10.1021/ja306856y

[bib21] Labib M Zamay AS Muharemagic D Chechik A Bell JC Berezovski MV 2012 Electrochemical sensing of aptamer-facilitated virus immunoshielding Anal Chem 84 1677 1686 22242920 10.1021/ac202978r

[bib22] Lichty BD Power AT Stojdl DF Bell JC 2004 Vesicular stomatitis virus: re-inventing the bullet Trends Mol Med 10 210 216 15121047 10.1016/j.molmed.2004.03.003

[bib23] Ebert O Harbaran S Shinozaki K Woo SL 2005 Systemic therapy of experimental breast cancer metastases by mutant vesicular stomatitis virus in immune-competent mice Cancer Gene Ther 12 350 358 15565179 10.1038/sj.cgt.7700794

[bib24] Ahmed M Cramer SD Lyles DS 2004 Sensitivity of prostate tumors to wild type and M protein mutant vesicular stomatitis viruses Virology 330 34 49 15527832 10.1016/j.virol.2004.08.039

[bib25] Obuchi M Fernandez M Barber GN 2003 Development of recombinant vesicular stomatitis viruses that exploit defects in host defense to augment specific oncolytic activity J Virol 77 8843 8856 12885903 10.1128/JVI.77.16.8843-8856.2003 PMC167243

[bib26] Stojdl DF Lichty BD tenOever BR Paterson JM Power AT Knowles S 2003 VSV strains with defects in their ability to shutdown innate immunity are potent systemic anti-cancer agents Cancer Cell 4 263 275 14585354 10.1016/s1535-6108(03)00241-1

[bib27] de Soultrait VR Lozach PY Altmeyer R Tarrago-Litvak L Litvak S Andréola ML 2002 DNA aptamers derived from HIV-1 RNase H inhibitors are strong anti-integrase agents J Mol Biol 324 195 203 12441099 10.1016/s0022-2836(02)01064-1

[bib28] Feng H Beck J Nassal M Hu KH 2011 A SELEX-screened aptamer of human hepatitis B virus RNA encapsidation signal suppresses viral replication PLoS One 6 e27862 22125633 10.1371/journal.pone.0027862 PMC3220704

[bib29] Sefah K Shangguan D Xiong X O'Donoghue MB Tan W 2010 Development of DNA aptamers using Cell-SELEX Nat Protoc 5 1169 1185 20539292 10.1038/nprot.2010.66

[bib30] Berezovski MV Lechmann M Musheev MU Mak TW Krylov SN 2008 Aptamer-facilitated biomarker discovery (AptaBiD) J Am Chem Soc 130 9137 9143 18558676 10.1021/ja801951p

[bib31] Barber GN 2005 VSV-tumor selective replication and protein translation Oncogene 24 7710 7719 16299531 10.1038/sj.onc.1209042

[bib32] Mallikaratchy PR Ruggiero A Gardner JR Kuryavyi V Maguire WF Heaney ML 2011 A multivalent DNA aptamer specific for the B-cell receptor on human lymphoma and leukemia Nucleic Acids Res 39 2458 2469 21030439 10.1093/nar/gkq996 PMC3064813

[bib33] Diallo JS Vähä-Koskela M Le Boeuf F Bell J 2012 Propagation, purification, and *in vivo* testing of oncolytic vesicular stomatitis virus strains Methods Mol Biol 797 127 140 21948474 10.1007/978-1-61779-340-0_10

[bib34] Labib M Zamay AS Muharemagic D Chechik AV Bell JC Berezovski MV 2012 Electrochemical differentiation of epitope-specific aptamers Anal Chem 84 2548 2556 22324738 10.1021/ac300047c

