# Supplementary material for: Aptamer-facilitated Protection of Oncolytic Virus from Neutralizing Antibodies
Source: Mol Ther Nucleic Acids. 2014 Jun 3;3(6):e167–. doi: 10.1038/mtna.2014.19 (PMC4078759; doi:10.1038/mtna.2014.19)
Supplement: Supplementary Figure S1 — Selection of anti-VSV aptamers. [file mtna201419x1.doc]

**Supporting Information**

**Aptamer-Facilitated Protection of Oncolytic Virus from Neutralizing Antibodies**

Darija Muharemagic^a^, Anna Zamay^a,b^, Shahrokh Ghobadloo^a^, Laura Evgin^c^, Anna Savitskaya^b^,

John C. Bell^c,d^ and Maxim V. Berezovski^a^

^a^ Department of Chemistry, University of Ottawa, 10 Marie Curie, Ottawa, Ontario K1N 6N5, Canada;

^b^ Institute of Molecular Medicine and Pathological Biochemistry, Krasnoyarsk State Medical University, 1 P. Zheleznyaka str., Krasnoyarsk, 660022, Russia;

^c^ Department of Biochemistry, Microbiology and Immunology, Faculty of Medicine, University of Ottawa, 501 Smyth Road, Ottawa, Ontario K1H 8L6, Canada;

^d^ Jennerex Inc., 450 Sansome Street, 16^th^ floor, San Francisco, California 94111, USA.

A

B

**Figure S1.** **Selection of anti-VSV aptamers.**

**(A)** Shielding aptamers against VSV were selected by eleven rounds of cell-SELEX procedure at 37^o^C which consisted of (1) four positive rounds of selection, (2) subsequent three rounds of negative selection against human blood cells, mouse blood cells and plasma, respectively, and (3) four rounds of competitive binding plate selection.

**(B)** Competitive binding plate selection (1) A non-labeled aptamer pool with an affinity for VSV was incubated in a 96-well plate to allow DNA to non-specifically adhere to the plastic. (2) Virus particles, previously incubated with an anti-VSV aptamer pool, were added to the well and bound to the adhered aptamer pool. (3) The sandwich-like complex of aptamers and virus was disrupted by addition of antibodies. Aptamers that were displaced from the surface of the virus were collected, amplified, and used for further selection.
