# Supplementary material for: Aptamer-facilitated Protection of Oncolytic Virus from Neutralizing Antibodies
Source: Mol Ther Nucleic Acids. 2014 Jun 3;3(6):e167–. doi: 10.1038/mtna.2014.19 (PMC4078759; doi:10.1038/mtna.2014.19)
Supplement: Supplementary Figure S2 — Gating strategy for the oncolytic virus for flow cytometry analysis. [file mtna201419x2.doc]

**Figure S2 Gating strategy for the oncolytic virus for flow cytometry analysis.** (**A**) A dot-plot of forward versus side scattering of debris from DPBS buffer. (**B**) Gating of VSV in DPBS (gate 1). Histograms of VSV (1 × 10^7^ PFU) with 100 nM FAM-labeled native ssDNA library (blue), 11^th^ aptamer pool (orange) and VSV alone (red).
