# Supplementary material for: Aptamer-facilitated Protection of Oncolytic Virus from Neutralizing Antibodies
Source: Mol Ther Nucleic Acids. 2014 Jun 3;3(6):e167–. doi: 10.1038/mtna.2014.19 (PMC4078759; doi:10.1038/mtna.2014.19)
Supplement: Supplementary Figure S3 — Determination of anti-VSV Ab concentration with 96-well plate assay. [file mtna201419x3.doc]

**Figure S3. (A)** Vero cell line, healthy (left) and infected by VSV (right), visualized by microscopy.

**(B)** Neutralizing cell assay done in a 96-well plate with YFP-expressing VSV (1x10^4^ PFU) and different anti-VSV nAbs concentrations. When neutralized, the virus is unable to proliferate inside the cell and thus the fluorescence will not be detected. Dilutions starting from 1/2000 and up were found to be effective in neutralizing the virus. The infection of cells was monitored with a FluorChem Q imaging system.
