# Supplementary material for: Aptamer-facilitated Protection of Oncolytic Virus from Neutralizing Antibodies
Source: Mol Ther Nucleic Acids. 2014 Jun 3;3(6):e167–. doi: 10.1038/mtna.2014.19 (PMC4078759; doi:10.1038/mtna.2014.19)
Supplement: Supplementary Figure S4 — Screening of anti-VSV aptamers in a 96-well plate. [file mtna201419x4.doc]

**Figure S4. Screening of anti-VSV aptamers in a 96-well plate.** YFP-VSV (1x10^4^ PFU) was incubated with different aptamers at 37^o^C for 1 hour. The coated virus was then added to a 1/2000 dilution of anti-VSV nAbs, incubated, and then added to a monolayer of Vero cells. The infection of cells was monitored with a FluorChem Q imaging system.
