# Supplementary material for: Aptamer-facilitated Protection of Oncolytic Virus from Neutralizing Antibodies
Source: Mol Ther Nucleic Acids. 2014 Jun 3;3(6):e167–. doi: 10.1038/mtna.2014.19 (PMC4078759; doi:10.1038/mtna.2014.19)
Supplement: Supplementary Figure S5 — Titration experiments showing the effect of different aptamer concentrations on VSV infectivity. [file mtna201419x5.doc]

10 1 10^-1^ 10^-2^ 10^-3^  10^-4^ 10^-5^  10^-6^  10^-7^  10^-8^  10^-9^  10^-10^ 10^-11^ 10^-12^

**Figure S5. Titration experiments showing the effect of different aptamer concentrations on VSV infectivity**. VSV and anti-VSV nAbs aptamer pools (concentration varying from 10 to 10^-12^ µM) were incubated with their respective targets and added to a monolayer of Vero cells. Percent of VSV infection was determined by standard plaque forming assay and compared to the positive control which consisted of 100 PFU’s. Each aptamer dose was done in triplicates.
