# Supplementary material for: Aptamer-facilitated Protection of Oncolytic Virus from Neutralizing Antibodies
Source: Mol Ther Nucleic Acids. 2014 Jun 3;3(6):e167–. doi: 10.1038/mtna.2014.19 (PMC4078759; doi:10.1038/mtna.2014.19)
Supplement: Supplementary Figure S6 — Real-time PCR (qPCR) showing degradation of monomeric, dimeric, and tetrameric aptamers. [file mtna201419x6.doc]

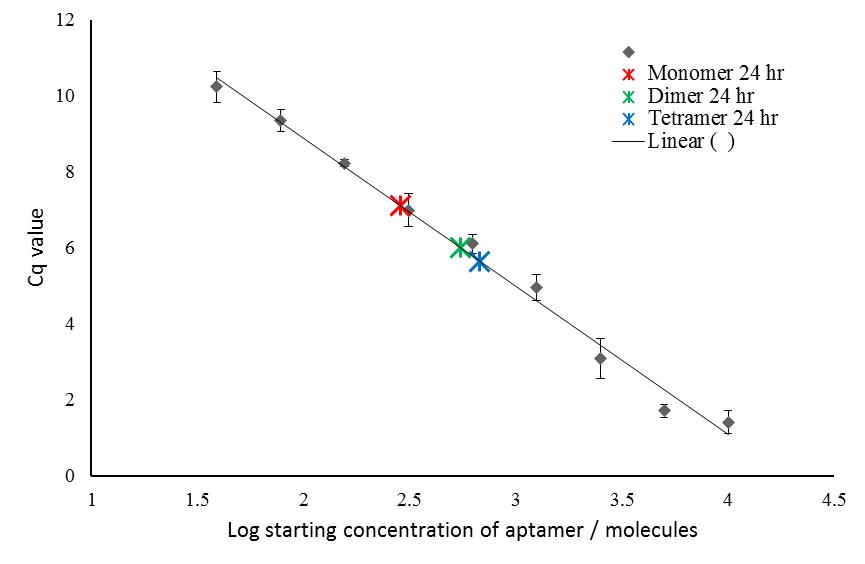


**A**

**B**


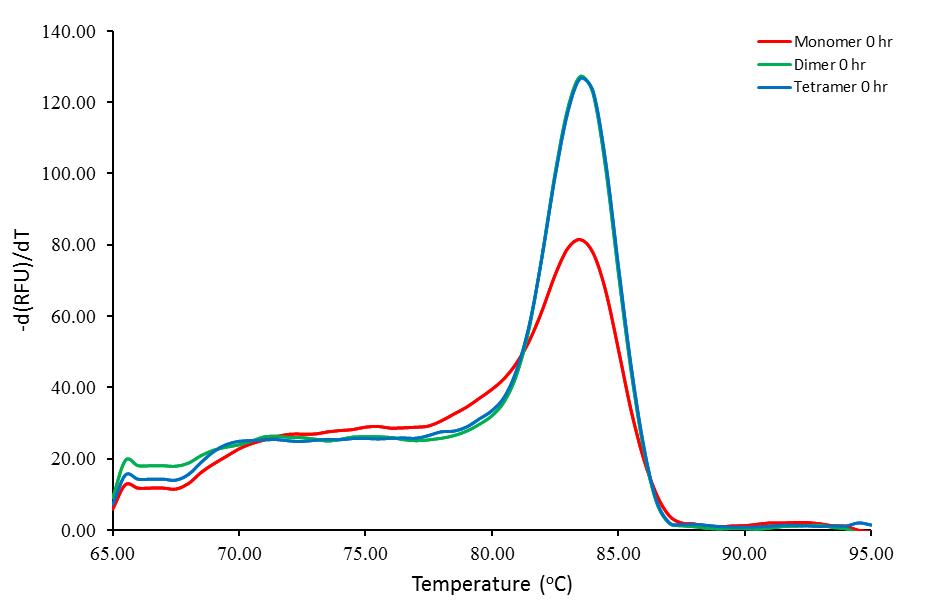

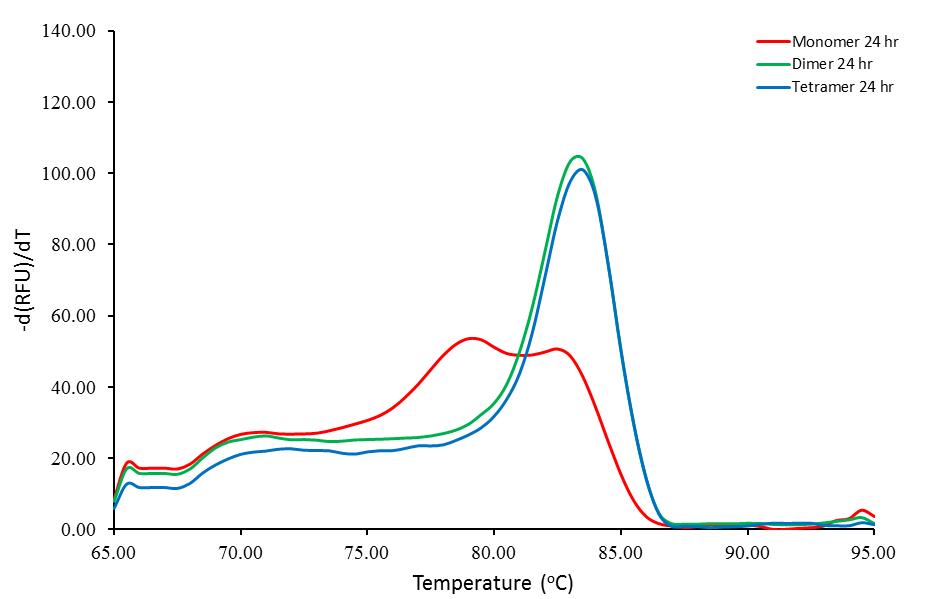


**Figure S6. Real-time PCR (qPCR) showing degradation of monomeric, dimeric and tetrameric aptamers.** Aptamers were incubated in serum and subjected to qPCR. (A) Standard curve showing the remaining concentration of different aptamer pools (initial concentration 10^4^ molecules); (B) melting curve of monomeric, dimeric and tetrameric aptamers before and after a 24-hour incubation in serum.
