# Supplementary material for: Aptamer-facilitated Protection of Oncolytic Virus from Neutralizing Antibodies
Source: Mol Ther Nucleic Acids. 2014 Jun 3;3(6):e167–. doi: 10.1038/mtna.2014.19 (PMC4078759; doi:10.1038/mtna.2014.19)

**Table S1. DNA sequences from competitive binding selection grouped into families of related sequences.** Aptamer sequences for VSV, where *f*: Ctc ctc tga ctg taa cca cg (the sequence of the forward PCR primer) and *cr*: gca tag gta gtc cag aag cc (the reverse-complement of the reverse PCR primer). Conserved nucleotides are in red; dashes are place holders for alignment. Sequence names starting with “M” are from the medium pool, whereas the ones designated with “S” sequences are from the strong pool.


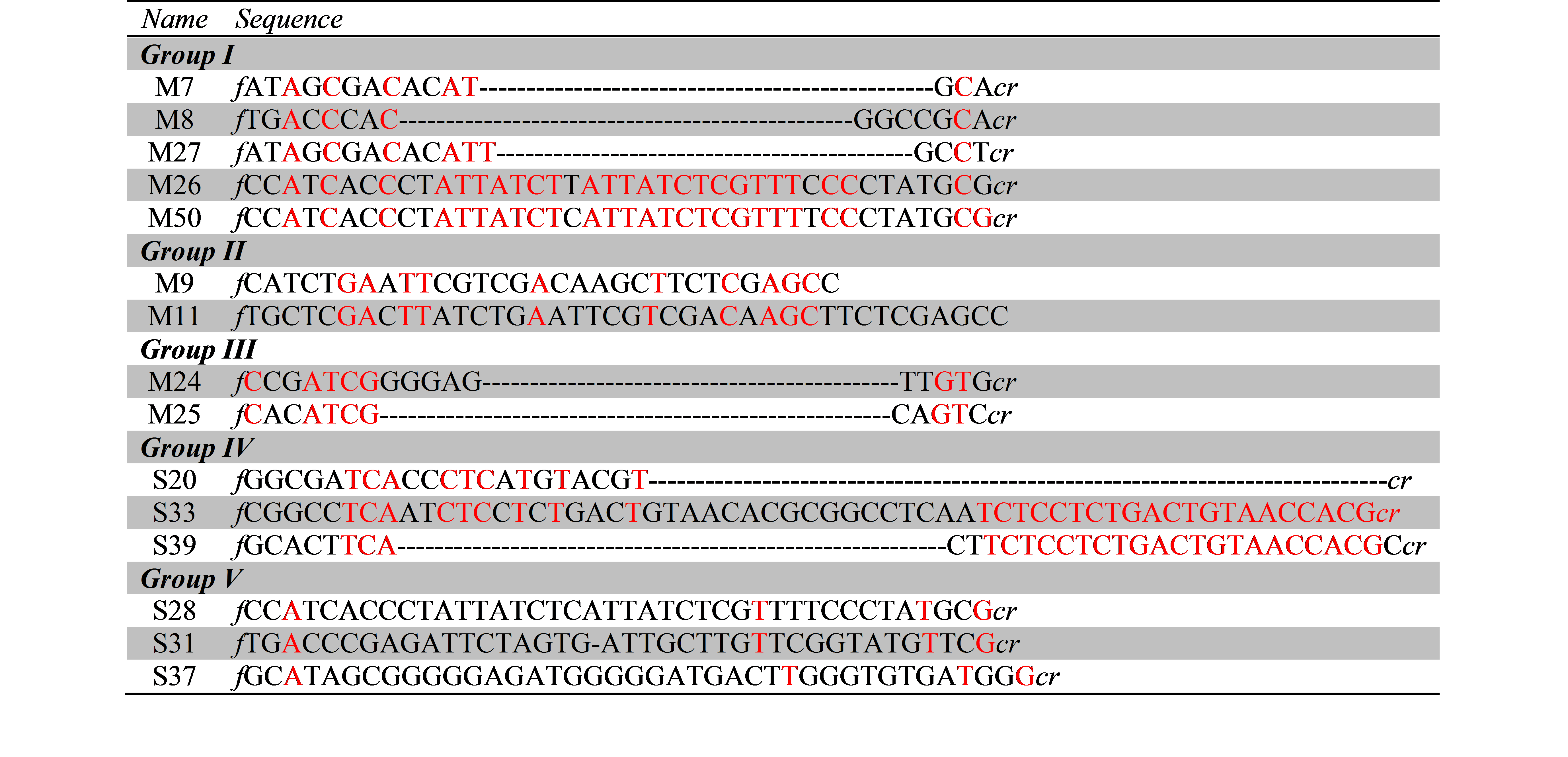

Supplement: Supplementary Table S1 — DNA sequences from competitive binding selection grouped into families of related sequences. [file mtna201419x7.doc]
